# Combining choline bitartrate and vitamin B12 ameliorates cognitive impairment in hypertensive elders with cognitive frailty

**DOI:** 10.1016/j.phrs.2024.107103

**Published:** 2024-02-07

**Authors:** Pasquale Mone, Valentina Trimarco, Urna Kansakar, Raffaele Izzo, Gaetano Santulli, Bruno Trimarco

**Affiliations:** Department of Medicine and Health Sciences “Vincenzo Tiberio”, University of Molise, Campobasso, Italy; Department of Medicine, Einstein Institute for Aging Research, Einstein Institute for Neuroimmunology and Neuroinflammation, Albert Einstein College of Medicine, New York, USA; Casa di Cura “Montevergine”, Mercogliano (Avellino), Italy ASL Avellino, Italy; Department of Neuroscience, Reproductive Sciences and Dentistry, “Federico II” University, Naples, Italy; Department of Medicine, Einstein Institute for Aging Research, Einstein Institute for Neuroimmunology and Neuroinflammation, Albert Einstein College of Medicine, New York, USA; Department of Advanced Biomedical Sciences, “Federico II” University, Naples, Italy; International Translational Research and Medical Education (ITME) Consortium, Academic Research Unit, Naples, Italy; Department of Medicine, Einstein Institute for Aging Research, Einstein Institute for Neuroimmunology and Neuroinflammation, Albert Einstein College of Medicine, New York City, NY, USA; Department of Advanced Biomedical Sciences, “Federico II” University, Naples, Italy; International Translational Research and Medical Education (ITME) Consortium, Academic Research Unit, Naples, Italy; Department of Molecular Pharmacology, Fleischer INstitute for Diabetes and Metabolism (FIDAM), Albert Einstein College of Medicine, New York City, NY, USA; Department of Advanced Biomedical Sciences, “Federico II” University, Naples, Italy; International Translational Research and Medical Education (ITME) Consortium, Academic Research Unit, Naples, Italy

**Keywords:** Aging, Choline, Cognitive impairment, Elderly, Frailty

Frailty is a worldwide increasing health problem leading to adverse outcomes such as cognitive impairment, particularly in older adults [1]. Cognitive frailty (CF) is a relatively novel type of frailty that refers to a condition characterized by the simultaneous presence of physical frailty and cognitive impairment, particularly in older adults [2]. CF represents a state of heightened risk for adverse health outcomes, including disability, dementia, falls, hospitalization, and mortality, compared to individuals who have either physical frailty or cognitive impairment alone. Mild cognitive impairment (MCI) is considered a transitional stage between normal cognitive aging and dementia. In elderly individuals, hypertension represents a typical comorbidity that further increases such risk, driving adverse events. Unfortunately, there is no accepted treatment for CF [3].

In a previous investigation, we evidenced the potential role of choline alphoscerate in frail hypertensive elders [4]. Choline bitartrate (CB) is one of the most common kinds of choline supplements and is known to be more absorbable than other forms [5]. Equally important, preclinical studies have reported a choline-sparing effect of Vitamin B12 (B12) supplementation, evidencing that patients with B12 deficit exhibit lower blood concentrations of choline [5]. On these grounds, we decided to test the effects of combining CB and B12 on cognitive impairment in hypertensive elders with CF. From January 2023 to December 2023, we evaluated 137 consecutive hypertensive elders with CF. We diagnosed CF in presence of MCI (defined by applying the Clinical Dementia Rating Scale, CDR: 0,5) and physical frailty (defined by the presence of at least 3 of 5 Fried Criteria [6]).

The study participants were recruited at the ASL (Local Health Authority of the Italian Ministry of Health) Avellino, Italy. Every patient or legally authorized representative signed a written informed consent. We performed this investigation following the ethical standards of the 2013 Declaration of Helsinki of the World Medical Association and in accordance with Good Clinical Practice guidelines. We obtained the approval by the Institutional Review Board of Napoli Centro. We evaluated global cognitive function at baseline and after 3 months using the MoCA test. MoCA scores range from zero to 30; a score of 26 and higher is considered normal.

All patients fulfilled the following criteria: confirmed diagnosis of hypertension and CF; age >65 years; no previous stroke and/or acute myocardial infarction, no coronary artery bypass grafting, EF >50%; Montreal Cognitive Assessment (MoCA) Score <26.

Of the screened patients, 27 did not fulfill the criteria and 11 did not give the consent to participate; a total of 99 patients successfully completed the study. We randomly assigned patients to CB + B12, Choline Alphoscerate, or no nutraceuticals. We followed-up each patient for 3 months, and at this time point we measured again the MoCA score, observing a beneficial effect of CB + B12 treatment on cognitive impairment (p < 0.001 vs baseline, Fig. 1). Patients receiving choline alphoscerate also presented significant salutary effects on cognitive impairment (p < 0.005 vs baseline). Yet, the difference at follow-up between the two treated groups was statistically significant, favoring CB + B12 (p < 0.05; Fig. 1).

Taken together, our data indicate that combining CB and B12 can improve global cognitive function in hypertensive patients with CF. Further investigations with a longer follow-up are necessary to confirm the results that we have obtained in elders with CF and hypertension as well as to extend our findings in other populations.

## Figures and Tables

**Fig. 1.**
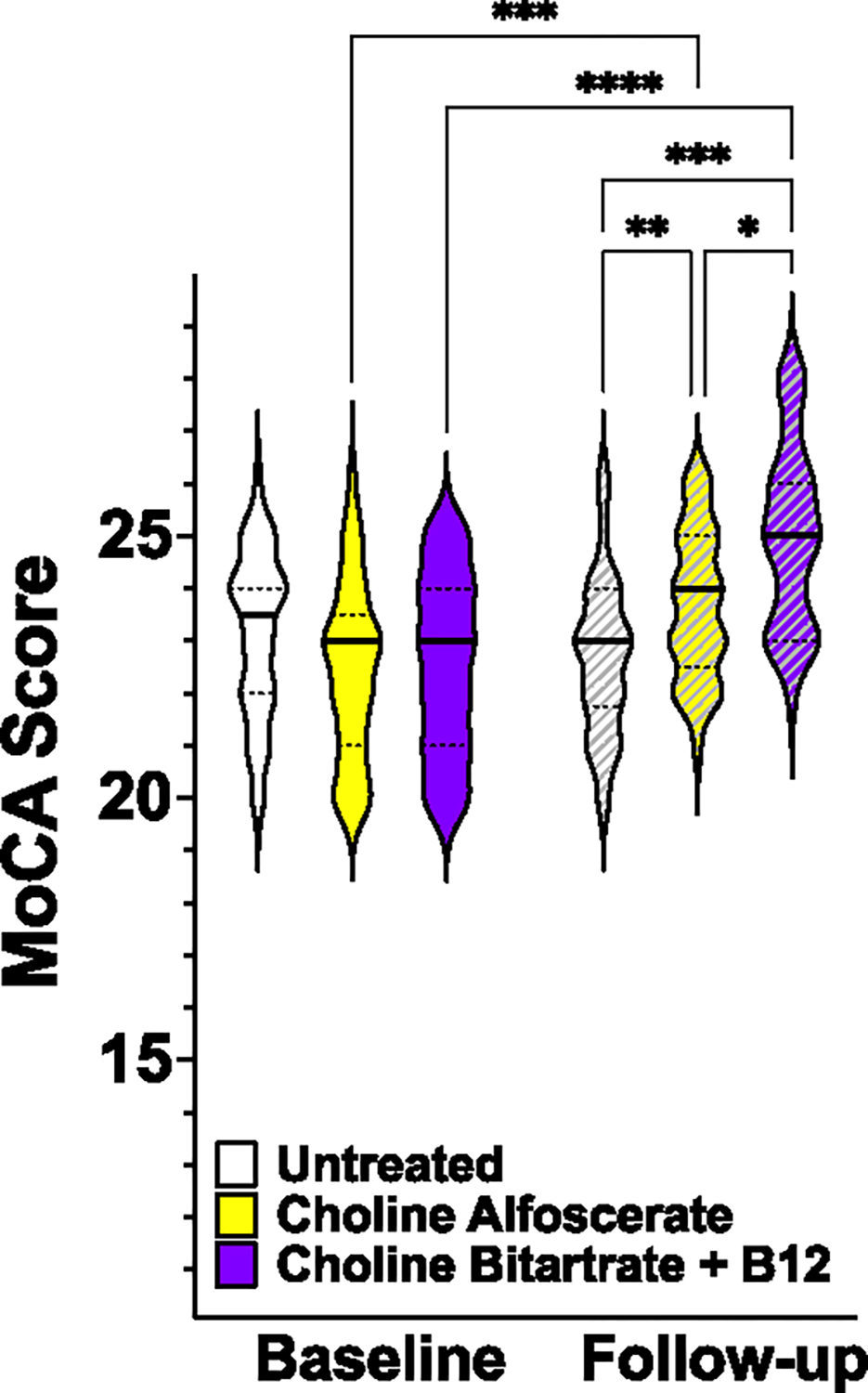
Montreal Cognitive Assessment (MoCA) Score assessed in the indicated groups at baseline and after 3 months. *: p < 0.05; **: p < 0.01; ***: p < 0.005; ****: p < 0.001 (ANOVA and Tukey-Kramer post hoc test).
